# Using of transporter proteins to improve the uptake efficiency of hydrophobic compounds by *Escherichia coli*: a coordinated synthesis of START protein and P450scc system proteins to enhance cholesterol biotransformation

**DOI:** 10.1186/s40643-025-00909-1

**Published:** 2025-07-21

**Authors:** Sofia V. Zamalutdinova, Ludmila V. Isaeva, Yaroslav V. Faletrov, Nikolay N. Eroshchenko, Alexey N. Kirushin, Vadim N. Tashlitsky, Mikhail A. Rubtsov, Ludmila A. Novikova

**Affiliations:** 1 Faculty of Biology, M.V. Lomonosov Moscow State University, Leninskie Gory, 1/12, Moscow, 119991 Russia; 2 Belozersky Institute of Physico-Chemical Biology, M.V. Lomonosov Moscow State University, Leninskie Gory 1/40, Moscow, 119991 Russia; 3 Research Institute for Physical Chemical Problems, Belarusian State University, Minsk, 220030 Belarus; 4 I.M. Sechenov First Moscow State Medical University, Moscow, 119991 Russia; 5 Faculty of Chemistry, M.V. Lomonosov Moscow State University, Moscow, 119991 Russia

**Keywords:** STARD1, STARD3, Heterologous expression, Cytochrome P450scc, Biotransformation of steroids

## Abstract

Synthesis of steroid drugs is possible through biotechnological methods, however, the low efficiency of transporting steroid substrates across microbial membranes is the challenge in using microorganisms for their production. STARD1 and STARD3 proteins (members of the START domain family) work in some mammalian cells in conjunction with the steroidogenic P450scc enzyme system (cytochrome P450scc, adrenodoxin reductase, and adrenodoxin), transporting cholesterol to the mitochondria, where cytochrome P450scc converts it into pregnenolone. In this study, we investigated the effect of STARD1 and STARD3 on P450scc efficiency in *E. coli* expression models. The combination of the STARD3(216–444) or STARD1(53–285) protein bearing an N-terminal periplasm-targeting sequence, with the P450scc system reconstituted in *E. coli*, resulted in an increase of pregnenolone synthesis by 3.2- and 1.9-fold, respectively, on a laboratory scale. STARD3(216–444) showed higher levels of expression and a greater effect on the incorporation of sterols into cells and whole-cell biotransformation of cholesterol, compared to STARD1(53–285). This study proposes a fundamentally new approach to optimizing the functioning of the P450scc system in microbial cells, which uses the cholesterol transfer protein to increase the uptake efficiency of a poorly soluble steroid substrate by bacteria. The demand for steroid medications is increasing, and the use of specific carrier proteins could be a useful tool to enhance the efficiency of whole-cell biosynthesis of various steroid compounds.

**Supplementary Information:**

The online version contains supplementary material available at 10.1186/s40643-025-00909-1.

## Graphical Abstract

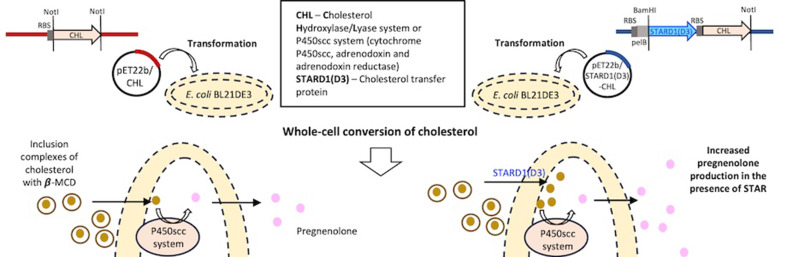

## Introduction

The use of natural or genetically modified microorganisms for the purposes of the biotechnology industry and medical research has become widespread. Cytochrome P450 monooxygenases (CYP450s) are involved in a variety of biosynthetic and metabolic processes, providing for chemo-, regio-, and stereospecific hydroxylation of different hydrophobic compounds. Data presented in the literature demonstrate the great potential of CYP450s to catalyse diverse reactions of steroid and sterol biotransformation in natural and transgenic microorganisms (Fernandes et al. 2003; Bureik and Bernhardt 2007; Do-nova and Egorova 2012; Girvan and Munro 2016). This is a prerequisite for the use of such microorganisms for the development of new biocatalytic processes for the production of commercially important steroid compounds, including widely used steroid pharmaceuticals (Hanlon et al. 2007; Novikova et al. 2009; Tong and Dong 2009; Urlacher and Girhard 2019; Li et al. 2020; Donova 2023). Of great practical interest for the synthesis of steroid drugs are, in particular, recombinant microorganisms that are capable of reproducing the processes occurring in steroidogenic organs of mammals, leading to the formation of the life-important steroid hormones.

In mammals, six cytochromes P450 (mitochondrial - P450scc (P450 *s*ide-*c*hain *c*leavage), P450c11, P450c18, and microsomal - P450c17, P450c21, P450c19) are involved in the sequential conversion of cholesterol to steroid hormones. These enzymes functioning concert with electron transfer proteins to form a complex P450 enzymatic system (Nebert et al. 2013). In 1989–1990, it was discovered that steroidogenic cytochromes P450 could function not only in mammalian cells, but also in microbial cells (Sakaki et al. 1989; Pompon et al. 1989; Slijkhuis et al. 1989; Hu and Chung 1990). This served as a significant catalyst for the development of research, aimed at obtaining diverse bacterial and yeast strains, encompassing steroidogenic proteins and functional mammalian P450 monooxygenase systems, which are involved in the cascade of steroidogenesis (e.g. see Szczebara et al. 2003; Kolar et al. 2007; Mauersberger et al. 2013; Zehentgruber et al. 2010; Gerber et al. 2015; Efimova et al. 2019a, b; Karpov et al. 2024). However, it should be noted that the catalytic activity of steroidogenic P450s expressed in microbial cells is usually low. One of the major problems is that the steroid substrates of cytochromes P450 are extremely hydrophobic and poorly soluble in aqueous media, which limits their uptake by the cell from the culture medium and, as a consequence, reduces the activity of the intracellularly localized enzyme. Various methods have been used to increase the solubility of steroids and the efficiency of their uptake by cells, such as the addition of organic additives to the culture media (Bertelmann et al. 2022; Zehentgruber et al. 2010; Efimova et al. 2019b) or the use of two-phase liquid systems (Liu et al. 1996; Braun et al. 2012), which may lead to impaired cell viability due to the effects on cell membranes (Thevenieau et al. 2010) or complicate technological processes. In addition, there is evidence that expression of heterologous genes encoding hydrophobic outer membrane pores can be used to improve steroid uptake by *E. coli* (Bertelman et al., 2022).

Recently, we proposed and tested a fundamentally new approach that could improve the efficiency of transferring exogenous hydrophobic substrates into host cells - the inclusion of a specific steroid carrier protein, StAR, into the cell membrane (Zamalutdinova et al. 2022).The StAR protein (Steroidogenic acute regulatory protein) also referred to as STARD1 (Lin et al. 1995), belongs to the so-called StAR-related lipid transfer (START) domain protein family, which is characterised by the presence of a relatively conservative lipid-binding domain (START domain, ~ 210 amino acids) (Ponting et al., 2022). STARD1 has been shown to mediate high-capacity cholesterol transfer in the mitochondria of mammalian steroidogenic cells (in adrenal glands, ovaries, testes, and brain) from the outer mitochondrial membrane to the inner membrane, where cytochrome P450scc (CYP11A1) is localized, catalyzing the initial reactions of cholesterol conversion in the steroidogenic cascade (Lin et al. 1995). STARD1 is imported into the mitochondrial matrix due to the presence of an N-terminal addressing presequence. However, N-terminally truncated STARD1 (residues 62–285), which includes only the functional START domain, loses the ability to be imported into mitochondria, but exhibits the same functional activity as the full-length protein (Arakane et al. 1996).

STARD3 is another member of the START protein family and a close homologue of the STARD1 (Moog-Lutz et al. 1997). The main function of STARD3 in mammalian cells is to implement of cholesterol transport at contact sites between endosomes and the endoplasmic reticulum (Wilhelm et al. 2017). STARD3 and STARD1 share similarities and differences. Both proteins contain a C-terminal lipid-binding domain capable of binding cholesterol (Moog-Lutz et al. 1997; Clark 2012), but they have different N-terminal sequences that target them to distinct subcellular compartments: mitochondria and late endosomes, respectively. These different localizations, in turn, suggest different roles for these proteins in cholesterol trafficking (Clark 2012; Larsen et al. 2020). However, there is evidence, that in the placenta, where STARD1 is absent (Lin et al. 1995), STARD3 is able to transfer cholesterol to the mitochondria and induce, like STARD1, the first step of steroidogenesis (Watari et al. 1997). Furthermore, Watari et al. demonstrated that a truncated version of the STARD3 protein, encompassing only the START lipid-binding domain (residues 235–445), can manifest functional activity at a level comparable to that of STARD1 (Watari et al. 1997).

Over the past decades, significant progress has been made in studying the function, catalytic and structural properties of STARD1 and STARD3 proteins. (Elustondo et al. 2017; Voilquin et al. 2019; Larsen et al. 2020). In the course of research, expression systems based on *E. coli* cells have been used to produce STARD1 and STARD3 for in vitro studies (e.g. see Arakane et al. 1996; Reitz et al. 2008; Sluchanko et al. 2016), antibody production (Moog-Lutz et al. 1997), and crystallization (Horvath et al. 2016). However, apart from our work on STARD1 (Zamalutdinova et al. 2022), no studies have been published on their function in living bacteria.

In a previous study, we demonstrated that recombinant *E. coli* BL21(DE3) cells synthesising a truncated version of human STARD1, which encompasses the functional domain (residues 53–285) and bears an N-terminal periplasmic targeting signal sequence (pelB), showed increased efficiency in the uptake of exogenous NBD-labelled cholesterol analogues compared to wild-type cells (Zamalutdinova et al. 2022). In this study, we investigated whether the cholesterol transport protein STARD3 (216–444) exhibits functional activity in bacterial cells. We also examined the impact of the human cholesterol transport proteins STARD1 and STARD3, targeted to the periplasm, on the efficiency of the cytochrome P450scc system of bovine adrenal cortex mitochondria, localized in bacteria.

The mammalian P450scc system (cholesterol side-chain cleavage system), including the monooxygenase cytochrome P450scc (cholesterol hydroxylase/C20-C22 lyase) and its redox partners adrenodoxin (Adx), a [2Fe-2S] ferredoxin, and NADPH-dependent FAD-containing reductase (AdR), converts of cholesterol to pregnenolone (3β-hydroxypre-5-en-20-one), a common precursor of glucocorticoids, mineralocorticoids, sex hormones, and neuroactive steroids (Nebert et al. 2013; Vallée 2016). Cytochrome P450scc is the sole protein that has the capacity to catalyse cholesterol side chain cleavage. The development of recombinant microorganisms with enhanced P450scc catalytic activity is of particular interest from a biotechnological point of view, as this will solve the major problem of synthesizing C21 steroid hormones and neurosteroids, which are used to produce many sought-after drugs (e.g. anti-inflammatory and anti-proliferative agents, drugs for the treatment of hormonal, reproductive and neurological disorders). The primary goal of the present study was to test the ability of human START transporter proteins to enhance the efficiency of cholesterol uptake by *E. coli* cells incorporating the P450scc system and thereby increase the efficiency of cholesterol conversion to pregnenolone by a recombinant strain.

## Materials and methods

### Materials

Isopropyl-β-D-thiogalactopyranoside (IPTG), δ-aminolevulinic acid (δ-ALA), cholesterol, pregnenolone, cholesterol oxidase (Sigma-Aldrich, USA), 22-NBD‐cholesterol (Thermo Fisher Scientific, USA), randomly methylated β-cyclodextrin (MCD) (Wacker Chemie, Germany), ZymoPURE™ Plasmid Miniprep Kit and Zymoclean™ Gel DNA Recovery Kit (Zymo Research, USA), goat primary anti-GFP antibodies conjugated with horseradish peroxidase (Rockland, USA; Item No. 600-103-215), horse-radish peroxidase-conjugated anti-rabbit antibodies (Sigma-Aldrich, USA), WesternBright™ ECL detection kit (Advansta, USA), Hybond-C Extra nitrocellulose filters (Amersham Biosciences, UK), DNA-modifying enzymes, DNA polymerases, and RNAse A (Thermo Fisher Scientific, USA) were used in the research. Oligonucleotides were synthesized by Evrogen (Russia). Primary antibodies (IgG-fraction) against bovine cytochrome P450scc, AdR, and Adx (Efimova et al. 2017) and 20-(NBD)amino-pregn-5-en-3β-ol (20NP) (Faletrov et al. 2017) were kindly provided by Prof. V. M. Shkumatov (Research Institute for Physical Chemical Problems, Belarusian State University, Minsk, Belarus).

### *E. coli* strains and plasmids

*E. coli* strains Top10 (Evrogen, Russia), BL21(DE3) (Gibco BRL, USA) and recombinant strains *E. coli*/pET-22b/STARD1, *E. coli*/pET-22b/STARD1-GFP (collection names) (Zamalutdinova et al. 2022), which were generated on the base of *E. coli* BL21(DE3) were used in this work. The bacterial vector pET-22b(+), containing the T7 bacteriophage promoter and a *pelB* sequence coding an N-terminal periplasm targeting signal of pectate lyase B from *Erwinia carotovora*, was from Novagen (USA). The RSFduet plasmid, which include cDNA for truncated human STARD3(216–444) (Tugaeva et al. 2020), was kindly provided by Dr. N. N. Sluchanko (Bach Institute of Biochemistry, Russian Academy of Science, Moscow, Russia). Plasmids pTrc99A/CHL (Efimova et al. 2019a), containing cDNAs for mature cytochrome P450scc, AdR, and Adx (1-108) from bovine adrenal cortex, pcDNA3.1/pCoxIV-P450scc-2A-GFP (Efimova et al. 2019b), encoding GFP, and pET-22b/STARD1 and pET-22b/STARD1-GFP (Zamalutdinova et al. 2022), including cDNA for truncated human STARD1(53–285) or STARD1(53–285)-GFP fusion protein, respectively, were constructed by us previously.

### Construction of plasmids

Cloning was performed using *E. coli* Top10 cells according to standard protocols (Sambrook and Russell 2001).

*pET-22b/STARD3*. A DNA fragment coding for STARD3(216–444) was excised with *BamH*I/*Not*I from the RSFduet-hSTARD3.216-444 and ligated into similarly digested vector pET22b(+). The intermediate vector was subsequently treated with *BamH*I and the Klenow fragment (in order to obtain the desired reading frame) and blunt-end ligated. The target plasmid, designated pET-22b/STARD3, comprises in-frame cDNA for the STARD3(216–444) protein, with an additional N-terminal pelB signal presequence (22 amino acids) (Fig. S1).

*pET-22b/*STARD3*-GFP*. cDNAs encoding STARD3(216–444) and GFP were PCR amplified and restriction cloned into the pET-22b(+) vector. The DNA fragment coding for STARD3 was obtained using the pET22b/STARD3 plasmid as a template and primers 1 (direct primer, containing *BamH*I site) and 2 (reverse primer, which introduces *Xba*I site and point nucleotide substitutions to remove the stop codon) (Table S1). The cDNA for GFP was amplified using pCoxIV-P450scc-2A-GFP and primers 3 and 4 (Table S1) to include flanking *Xba*I/*Not*I restriction sites. PCR products cleaved with *BamH*I/*Xba*I (*STARD3* gene) or *Xba*I/*Not*I (*GFP* gene) were inserted into the *BamH*I*/Not*I digested vector pET-22b(+). The resulting plasmid contains a sequence that encodes the STARD3 protein, fused to the N-terminal pelB sequence, and the C-terminal GFP protein in one reading frame (Fig. S1). *pET22b/STARD1-CHL.* A PCR fragment including cDNA for P450scc-RBS-Adx-RBS-AdR, which is referred to as CHL, and the preceding RBS sequence, was obtained using pTrc99A/CHL as a template and primers 5 and 6 (Table S1), both containing a unique restriction site *Not*I. Ligation of the fragment encoding RBS-CHL into the *Not*I-treated vector pET22b/STARD1 resulted in pET22b/STARD1-CHL comprising a polycistronic cassette with hybrid cDNA encoding STARD1(53–285) bearing target sequence pelB, and the mature forms of bovine P450scc, AdR and Adx (Fig. S1). After cell transformation, clones with the insert in the correct orientation were selected using PCR screening with primers 7 and 8 (Table S1), matched the middle of the AdR protein nucleotide sequence and the end of the ampicillin resistance gene, respectively. This allowed to obtain the desired 1.9 kb product only if the insert was oriented correctly. The *pET22b/STARD3-CHL* plasmid was constructed in the same way as pET22b/STARD1-CHL (see above), but using the base vector pET22b/STARD3. Plasmid constructs were verified by restriction analysis and Sanger automatic DNA sequencing.

### Expression of Recombinant proteins

Expression of heterologous genes was performed by growing cells using two approaches. *In one case* gene expression was induced by adding of the transcription inducer IPTG (0.5 mM) to the TB growth medium containing ampicillin (100 µg/ml) and δ-ALA (0.5 mM) as an additional source of heme, and the cells were grown with shaking (180 rpm) for 17–48 h at 25–37 °C (Efimova et al. 2019b). *In another case*, recombinant *E. coli* cells were grown in autoinduction medium (LB medium with added salts and sugars) (Studier 2005) containing 100 µg/ml ampicillin and δ-ALA (0.5 mM). Expression of heterologous genes was induced by 0.2% α-lactose contained in the culture medium; cells were incubated for 24 h at 37 °C. The conditions of the experiments conducted in this study are summarised in Supplementary Table S2.

### Analysis of cells for GFP fluorescence

The analysis was performed as previously described (Zamalutdinova et al. 2022). *E. coli* cells were cultivated in the presence of IPTG for the induction of heterologous genes expression (*see Sect.* Expression of Recombinant proteins) for 17 h. Cells were pelleted by centrifugation (12000 g, 5 min), and resuspended in the buffer (10 mM Tris-HCl (pH 8.0), 150 mM NaCl) to obtain suspensions with the absorbance А_600_ = 1.5. The resulting suspensions were analyzed for the presence of GFP using a FluoroMax-3 fluorimeter (HORIBA Jobin Yvon GmbH, Germany) with excitation at 395 nm and emission at 507 nm (slit width, 5 nm).

### SDS-PAGE and Western immunoblotting

Lysates of *E. coli* cells were analyzed using SDS-PAGE (Laemmli 1970) followed by Western blotting (Towbin et al. 1979). Bacteria were pelleted by centrifugation of 1.5 ml of culture (12000 g, 5 min; Eppendorf MiniSpin, Germany), resuspended in 100 µl of the buffer to prepare the samples for electrophoresis (Laemmli 1970) and disrupted by boiling. 15–20 µl of samples containing 1.5–60 µg of total protein, were loaded into each lane. Protein concentration was determined according to Lowry et al. (Lowry et al. 1951). When testing for the presence of STAR-GFP, the membranes with transferred proteins were treated with the conjugate of primary goat anti-GFP antibodies with horseradish peroxidase at 1:10000 dilution (V/V), and ECL substrate according to the instructions of the manufacturers. When testing for the presence of P450scc system proteins, blots were decorated with the primary rabbit polyclonal antibodies against P450scc (6.0 mg/ml), AdR (9.5 mg/ml), or Adx (2.5 mg/ml) at 1:7500, 1:7500, and 1:4500 dilution (V/V), respectively, and then were treated with the conjugate of anti-rabbit secondary antibodies with horseradish peroxidase (1 mg/ml) at 1:15000 dilution (V/V) and ECL substrate. Densitometric analysis of immunospecific bands was performed using the ChemiDoc™ MP imaging system (Bio-Rad, USA) and Image Lab software (Bio-Rad, USA). The calculated amount of heterologous protein in the samples was normalized to 1 µg total protein.

### Analysis of STARD1 and STARD3 functional activity

The ability of carrier proteins to transport fluorescent cholesterol analogs into cells was estimated from the efficiency of 22-NBD-cholesterol and 20NP accumulation in *E. coli*/pET22b/STARD1, *E. coli*/pET22b/STARD3 and non-transformed cells, as described previously (Zamalutdinova et al. 2022). Cells were cultured for 24–48 h in the presence of lactose and 22-NBD-cholesterol (10 µM) or 20NP (4 µM) under conditions that allowed expression of STAR proteins. Culture aliquots were collected at specified time intervals. Then, taking into account the A_600_ values in the cultures, samples were taken for the study, containing an equal number of cells. The cells were washed with MCD solution, resuspended in 96% ethanol and disrupted using ultrasound. The lysates were analyzed for the content of fluorescent steroids using a FluoroMax-3 fluorimeter with excitation at 470 nm and emission at 535 nm (slit width, 5 nm). The R programming language (R. Core Team, 2021) was used for the data analysis and the graphics.

For the analysis of STARD1 and STARD3 activity towards cholesterol *E. coli* strains were cultured under conditions of induction of heterologous protein synthesis in the presence of IPTG (*see Sect.* Expression of Recombinant proteins). Cholesterol was added to the cultivation medium simultaneously with the inducer to a final concentration of 0.5 mM, in the form of a 100-fold aqueous concentrate as a solution in MCD (250 mM). Cells were incubated for 24 h at 25 °C and 180 rpm. Aliquots of the cultures were collected (3 ml; A_600_ was about 23), the cells were pelleted by centrifugation (12000 g, 5 min), washed with 0.05 mM MCD solution (twice) and suspended in 1 ml of water. Organic substances were extracted from the suspensions with ethyl acetate (twice with 0.5 ml). The organic phases were combined and vacuum-evaporated to dryness, the residue was redissolved in 0.25 ml of 96% EtOH and the insolubles formed were separated by centrifugation (5000 *g*, 15 min). Analysis of the cholesterol content in the samples (as ethanolic solutions) was performed using HPLC-MS/MS. Chromatographic separation was carried out on an ExionLC chromatograph (Sciex, Canada) using a Kinetex C8 column (30 × 2.1 mm, 1.7 μm; Phenomenex, USA).The elution was performed at 0.4 ml/min; 35 °C in the isocratic mode with 15% of phase A (H_2_O with the addition of 0.1% formic acid), 85% of phase B (acetonitrile with the addition of 0.1% formic acid); injection volume − 1 µl. Detection was performed on a Scix 4500 QTRAP mass-spectrometer (Scies, Canada) using chemical ionization at atmospheric pressure in positive mode (APCI+; MRM mode: m/z 369.1→161.2). Ionization source parameters: curtasin gas – 35; nebulizer current – 3, 400 °C; ion source gas 1–50; collision gas – medium. Parameters of the mass detector: DP − 80; EP − 10; CE − 30; CXP- 12. Processing of the results was performed using Analyst 1.6.3 software and MultiQuant 3 (Scies, Canada). The amount of steroid present in the sample was estimated by the external standard method with a cholesterol control sample (the calibration range 0.53–200 µg/ml) and normalized to the number of cells in the culture at an optical density of 1.0.

### Measuring of cholesterol hydroxylase/lyase activity of *E. coli* cells in vivo

The efficiency of cholesterol transformation by recombinant cells was determined as previously described (Efimova et al. 2019b). Briefly, heterologous expression was carried out in the presence of IPTG (*see Sect.* Expression of Recombinant proteins) for 22 h and the cell suspension (50 ml, А_600_ = 0.34) was concentrated in the 50 mM sodium phosphate buffer (pH 7.4) to 25 g wet weight/l. Resting cells were cultured in the presence of IPTG (1 mM), δ-ALA (0.5 mM), ampicillin (50 µg/ml), glycerol (2.0%, *V*/*V*), MCD (2.5 mM), and cholesterol (0.5 mM) for 24 h (180 rpm, 25 °C). Aliquots of the culture medium, obtained from the cultures (5 ml), were collected, the organic substances were extracted, and the extracts were evaporated. The resulting precipitates were dissolved in 96% ethanol (300 µl), the formed insolubles were separated by centrifugation and pregnenolone content in the obtained samples was determined by high-pressure liquid chromatography (HPLC) using the HPLC system Series 1200 (Agilent, USA) as previously described (Efimova et al. 2019a) with a pregnenolone control sample. Calibrations were performed by the external standard method based on peak areas. The standard curve was linear from 1 to 100 µg/ml for pregnenolone.

In other experiments, the precipitates, obtained as described above, were suspended in 200 µl of 135 mM Na-phosphate buffer (pH 7.4), containing 0.05% Tween 20 (V/V), and treated with cholesterol oxidase (1 U; 37 °C, 1 h) to oxidize formed pregnenolone to progesterone. Progesterone content was determined by enzyme-linked immunosorbent assay (ELISA) using the immunoassay kit “IFA PROGESTERONE” (Xema, Russia) based on the use of antibodies to progesterone, according to the manufacturer’s protocol. Samples obtained from non-transformed cells, which did not express cytochrome Р450scc were used as a negative control. The values obtained were normalized to 1 L of suspension of resting cells. The R programming language (R. Core Team, 2021) was used for the data analysis.

## Results and discussion

### STARD3 expression analysis

In this study, the first task was to determine the characteristics of STARD3 expression and function in *E. coli* cells and to compare them with similar characteristics of STARD1. We developed a bacterial cell model in which synthesized STARD3 was directed to the periplasmic space, similar to STARD1, which is synthesized in *E. coli* strains that we have previously obtained (Zamalutdinova et al. 2022). We used cDNA encoding only the functional START domain of STARD3 (residues 216–444), as in our previous work with STARD1(53–285) (Zamalutdinova et al. 2022). For convenience, STARD3(216–444) and STARD1(53–285) will be referred to as STARD1 and STARD3, respectively, in the text.

To analyze the expression of STARD3, we used *E. coli* cells transformed with the pET22b/STARD3-GFP plasmid, which contains cDNA encoding STARD3 with an N-terminal pelB signal peptide and the reporter protein GFP fused to its C-terminus. The advantage of fusing with GFP is that it allows the identification of heterologous proteins and monitoring of their content in recombinant cells using fluorescence detection techniques and Western immunoblotting with GFP-specific antibodies.

**Fig. 1:**
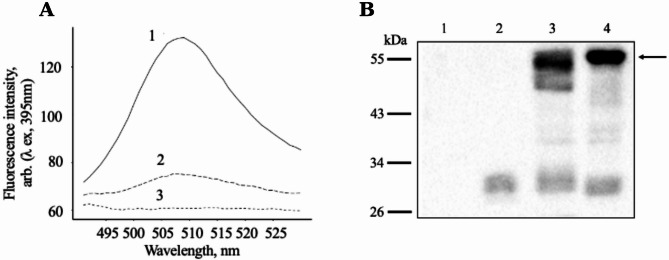
Expression of STARD3-GFP and STARD1-GFP in *E. coli* cells. **(A)** Analysis of *E. coli*/pET22b/STARD3-GFP (1), *E. coli*/pET22b/STARD1-GFP (2), and control cells (3) for GFP fluorescence; induction for 17 h, A_600_ = 1.5; fluorescence maximum – 507 nm. **(B)** SDS-PAGE (10% gel) and Western blotting analysis using anti-GFP antibodies; lanes: 1, control cell lysate (10 µg); 2, GFP standard (0.25 µg); 3, *E. coli*/pET22b/STARD1-GFP cell lysate (10 µg); 4, *E. coli*/pET22b/STARD3-GFP cell lysate (1.5 µg). The arrow indicates the position of STARD3-GFP protein. The molecular weights of standard protein markers (kDa) are shown on the left

The analysis of the GFP fluorescence of cell preparations obtained as described in the “Materials and Methods” (*see Sect.* Analysis of cells for GFP fluorescence) using fluorescence spectroscopy showed that, in contrast to non-transformed control cells, *E. coli*/pET22b/STARD3-GFP cells exhibited a spectrum corresponding to correctly folded GFP chromophore with an emission maximum fluorescence (507 nm), indicating the presence of a full-sized fused protein (Fig. 1A). A quantitative assessment of the content of fluorescent proteins in preparations of recombinant cells was conducted, revealing that the fluorescence intensity recorded in *E. coli*/pET22b/STARD3-GFP cells is significantly higher than in *E. coli*/pET22b/STARD1-GFP cells (approximately 3-fold for cells grown under induction conditions for 17 h; data from three independent experiments).

Furthermore, a significant difference in the levels of STARD3-GFP and STARD1-GFP fluorescent proteins within cells was evident when the GFP fluorescence emission was detected by UV excitation. As demonstrated in Fig. S2, when cell suspensions with equivalent optical density (A_600_) were evaluated for fluorescence at 366 nm UV excitation, the fluorescence intensity of cells synthesizing STARD3-GFP was considerably higher than that of cells synthesizing STARD1-GFP.

Western blot analysis using anti-GFP antibodies (*see Sect.* SDS-PAGE and Western immunoblotting) confirmed that the full-length STARD3-GFP protein with the expected molecular weight (~ 57 kDa) is present in the lysate of *E. coli*/pET22b/STARD3-GFP cells (Fig. 1B, lane 4). This analysis also showed that the STARD3-GFP fusion protein is present at significantly higher levels in recombinant *E. coli* cells than the STARD1-GFP protein (see Fig. 1B, lanes 4 and 3; 1.5 µg and 10 µg of total protein were loaded per lane, respectively). Furthermore, it showed that STARD3-GFP is more stable than STARD1-GFP. Immunoblotting revealed the presence of a smaller band in addition to the full-length STARD1-GFP (Fig. 1B, lane 3), with 65.9 ± 4.7% of the total STARD1-GFP being a full-length protein detected in the upper band with a molecular mass of ~ 54 kDa. Densitometric analysis of immunospecific bands indicates that the content of STARD3-GFP in *E. coli* cells exceeds that of full-length STARD1-GFP by approximately 8 ± 3 times (mean value ± SD of 4 independent experiments). This difference may be due to variations in their synthesis rates, or to the partial proteolysis of the STARD1-GFP protein, as indicated by the presence of a smaller protein fragment.

**Fig. 2:**
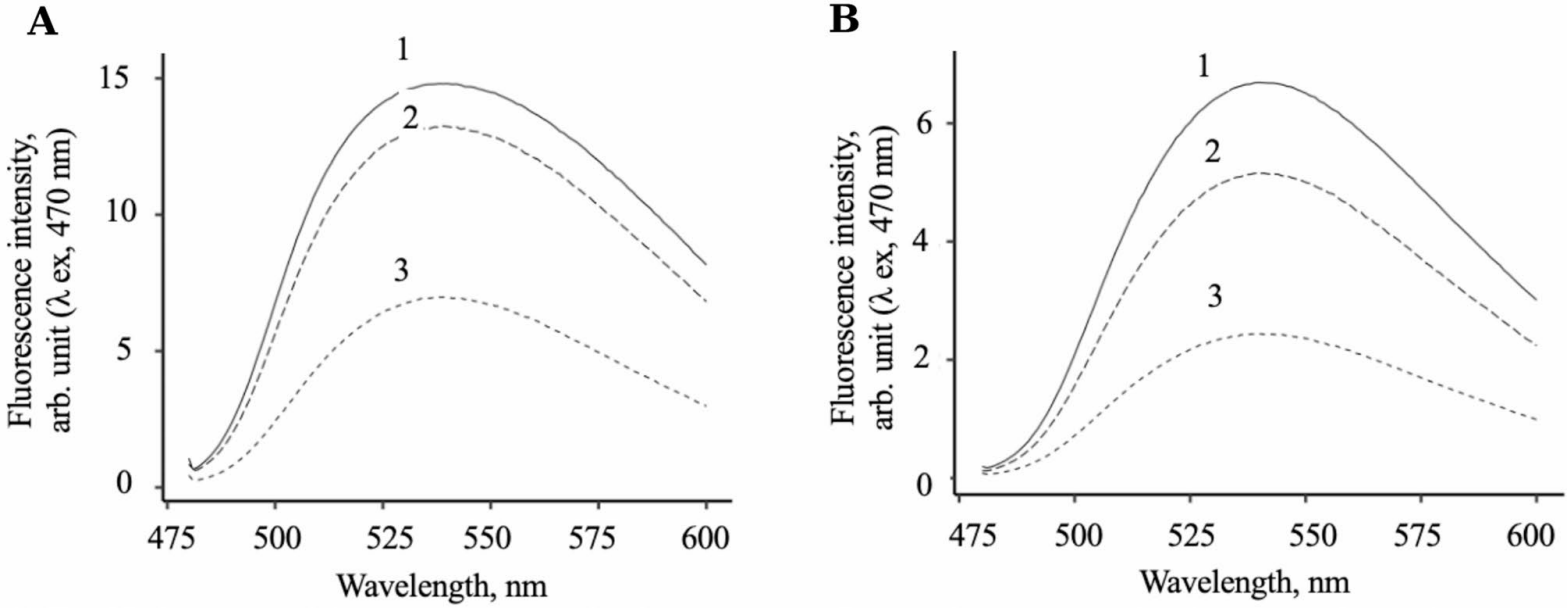
Effect of STARD3 and STARD1 on the incorporation efficiency of fluorescent sterols into bacterial cells. Analysis of the content of fluorescent cholesterol analogs 22-NBD-cholesterol (**A**) and 20NP (**B**) in *E. coli* cells. The fluorescence spectra of preparations obtained from *E. coli*/pET22b/STARD3 (1), *E. coli*/pET22b/STARD1 (2), and control cells (3); induction for 24 h in the presence of NBD-labeled sterols. Fluorescence maximum, 530 nm

In line with previous studies (Waldo et al. 1999), if the upstream protein in the fusion polyprotein (STARD3 in this case) does not fold correctly, the GFP reporter protein will also fail to fold correctly and will not exhibit the characteristic spectrum. Consequently, STARD3-GFP is synthesized in bacteria (Fig. 2B) and, moreover, STARD3 adopts the correct conformation.

It can be noted that in addition to STARD-GFP fusion proteins, a protein with a molecular mass equivalent to GFP is also detected in recombinant cell lysates (Fig. 1B). The extant literature contains evidence indicating that some GFP-containing fusion proteins expressed in *E. coli* could be subjected to partial proteolytic degradation, resulting in the formation of a protein with a molecular mass and immunospecificity corresponding to that of GFP (see, for example, Wu et al. 2000; Shi and Su, 2001). In our case, the relatively small portion of GFP fragments indicated that the majority of the STAR-GFP fusion proteins had not undergone proteolytic degradation. Perhaps that the proteolysis of fusion proteins occurs due to the linker region connecting the formed protein domains (in this case, STAR and GFP) being less stable and more accessible to cellular proteases.

### Analysis of the functional activity of STARD3

Experiments to evaluate the effect of STARD3 on the efficiency of steroid assimilation by *E. coli* cells were carried out using cells transformed with the plasmid pET22b/STARD3, which directs the synthesis of STARD3 with the N-terminal pelB sequence but lacks the C-terminal GFP to exclude the possibility of its influence on the function of the carrier protein.

In the first set of experiments, we used the fluorescent analogs of cholesterol 22-NBD-cholestrol and 20NP as steroid substrates. Previously, it was reported that purified STARD1 and STARD3 were able to bind 22-NBD-cholesterol and 20NP in vitro (Tugaeva et al. 2018), and that these steroids could penetrate into *E. coli* cells (Efimova et al. 2015). Cultures of recombinant *E. coli*/pET22b/STARD3 cells, control non-transformed cells, and *E. coli*/pET22b/STARD1 cells (Zamalutdinova et al. 2022), which were included in the experiment to compare the activities of STARD3 and STARD1, were grown under induction conditions in the presence of 22-NBD-cholesterol or 20NP for 24 and 48 h. As established by fluorimetry (*see Sect.* Analysis of STARD1 and STARD3 functional activity), following cultivation of cells in the presence of NBD-labeled cholesterol analogues, the sterol content in *E. coli*/pET22b/STARD3 was higher than that observed in control cells at both designated time points. As an example, Fig. 2 shows the result of the accumulation of steroid compounds in *E. coli* cells during 24 h of cultivation.

As shown in the data presented, the content of 22-NBD-cholesterol and 20NP in *E. coli*/pET22b/STARD3 cells was approximately 2 times higher (Fig. 2A) and approximately 2.6 times higher (Fig. 2B), respectively, in comparison with that observed in control cells. Furthermore, STARD3 to exhibits a higher activity towards NBD-labeled cholesterol analogues in comparison to STARD1. According to the results of all experiments performed (9 measurements for each substrate in 3 independent experiments), the presence of STARD3 in cells increases the efficiency of sterol uptake compared to control cells by 1.5 times for 22-NBD-cholesterol (95% С.I.[1.2–1.8]) and by 3 times for 20NP (95% С.I.[2.1-4.0]), and its activity exceeds that of STARD1 by approximately 1.2 and 2.0 times, respectively.

In the next step, we tested the possibility of using human STARD3 and STARD1 to increase the efficiency of cholesterol uptake by bacterial cells (*see Sect.* Analysis of STARD1 and STARD3 functional activity). The control strain and the recombinant *E. coli*/pET22b/STARD3 and *E. coli*/pET22b/STARD1 strains were grown under induction conditions in the presence of 0.5 mM cholesterol. The cholesterol content in organic extracts obtained from the studied cells was determined by HPLC method coupled with mass spectrometry. Figure S3, which shows fragments of HPLC-MS profiles of the obtained samples, illustrates the difference in cholesterol accumulation efficiency between non-transformed and recombinant *E. coli*/pET22b/STARD1 and *E. coli*/pET22b/STARD3 cells (Fig. S3 A, B,C, respectively). After 24 h of incubation, the cholesterol content in cells containing either the STARD3 or STARD1 protein was found to be 1.62 ± 0.28 and 1.34 ± 0.70 times higher, respectively, than in the control cells (per number of cells in 1 ml of culture at an optical density of 1.0; mean ± SD; *p* < 0.05) (Table S4).

In general, these experiments demonstrated that STARD3 is able to display functional activity in living bacteria similar to that of STARD1 - to transfer sterols across the cell membrane and thereby facilitate their uptake by cells. Besides, the activity of STARD3 towards used sterols exceeds the activity of STARD1. Compared to the wild-type strain, recombinant cells expressing STARD3 absorb sterols more efficiently, by approximately 1.5, 3.0 and 1.6 times for 22-NBD-cholesterol, 20-NP and cholesterol, respectively, and compared to *E. coli*/pET22b/STARD1, the increase is approximately 1.2, 2.0 and 1.2 times, respectively.

According to the data obtained (Fig. 2), 22-NBD-cholesterol and 20NP are incorporated into the cell with different efficiencies in both the presence and absence of STAR proteins. This may be due to differences in physicochemical properties of these steroids. The difference in sterol uptake efficiency between cells expressing STARD3 and those expressing STARD1 could be due to different affinities of these proteins for cholesterol and its analogues. In particular, it has been reported that the START domain of STARD3 has a slightly higher affinity for 22-NBD-cholesterol in vitro than the START domain of STARD1 (Reitz et al. 2008). It has also been reported that the position of the NBD group affects the interaction of NBD-labelled cholesterol analogues with the STARD1 protein (Tugaeva et al. 2018). Moreover, the difference in the efficiency of steroid accumulation in cells expressing STARD1 and STARD3, may be due to the differences in the expression levels of recombinant proteins.

Human STARD3 and STARD1 are of significant importance as regulatory proteins, controlling vital functions in mammals and being involved in the development of severe disorders. Despite many interesting studies concerning these proteins, the detailed mechanisms underlying their function are still far from being completely clear (Clark 2012; Tugaeva et al. 2018; Voilquin et al. 2019). Based on the data indicating that STARD3 and STARD1 are able to transport cholesterol in different types of mammalian tissues (e.g. see Lin et al. 1995; Arakane, 1996; Clark 2012; Elustondo et al. 2017), and the results obtained, it can be assumed that the mechanisms of these proteins’ function can be implemented not only in different types of mammalian cells, but possibly also in microbial cells. The functioning of STARD3 and STARD1 in *E. coli* cells opens up prospects for using these bacteria as a simple model system for studying their characteristics.

### Co-expression of the STARD1 or STARD3 gene with genes of the P450scc system proteins

**Fig. 3:**
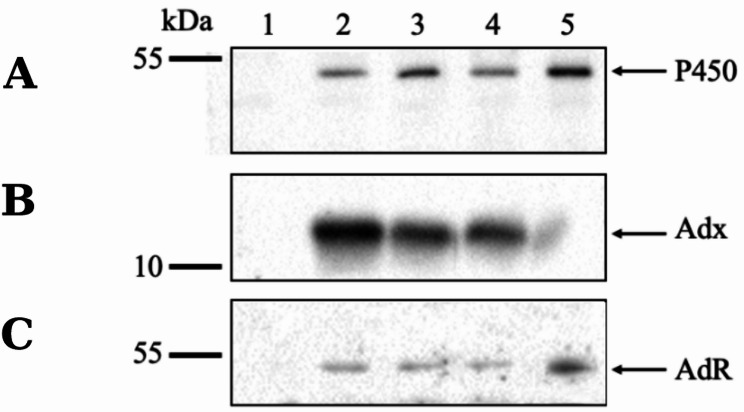
Co-expression of bovine P450scc, Adx, and AdR in IPTG-induced *E. coli* cells. Western blotting of cell lysates after SDS-PAGE in 10% (**A**) or 15% (**B**, **C**) gel followed by immunostaining with antibodies against P450scc (**A**), Adx (**B**), or AdR (**C**). Lysates of *E. coli* cells transformed with plasmid pET22b/CHL (2), pET22b/STARD1-CHL (3), or pET22b/STARD3-CHL (4). Non-transformed *E. coli* cell lysate (1) was used as a negative control. Lysate of *E. coli*/pBar_Triple cells (Efimova et al., 2019a) (5) synthesizing three separate mature proteins of the P450scc system was used as positive control. Total protein loading/lane: **A**, lines 1–4, 5: 3 µg and 1 µg, respectively; **B**, lines 1–4, 5: 60 µg and 20 µg, respectively; **C**, lines 1–4, 5: 30 µg and 20 µg, respectively. Molecular weights of standard marker proteins (kDa) are shown on the left

The next task of the study was to evaluate the influence of human cholesterol transporter proteins on the efficiency of cholesterol transformation by cytochrome P450scc present in *E. coli* cells. For this purpose, it was supposed to obtain a functionally coupled system in *E. coli* cells, including proteins of the P450scc (cholesterol hydroxylase/lyase, CHL) system (P450scc, Adx, and AdR) and STARD cholesterol transporter protein. The co-expression of four or three genes was carried out using a single polycistronic plasmid, which greatly facilitated the construction of recombinant strains. To implement co-expression of P450scc system proteins and STARD1 or STARD3 in bacteria, we generated vectors pET22b/STARD1-CHL and pET22b/STARD3-CHL. Additionally, we obtained the pET22b/CHL vector for co-expressing only cDNAs that encode P450 system proteins (*see Sect.* *E. coli* strains and plasmids, Fig. S1). These polycistronic vectors include heterologous genes separated by ribosome binding sites (RBS) and controlled by a single promoter. In this case, transcription results in the formation of a single hybrid mRNA with independent translation of individual proteins. Here, we used cDNAs encoding the mature forms (without mitochondrial targeting sequences) of bovine cytochrome P450scc, Adx, and AdR, as mature proteins can form an active enzymatic P450scc system in microbial cells (Szczebara et al. 2003; Novikova et al. 2009). All constructed plasmids successfully direct the synthesis of the P450scc system proteins. Full-length mature proteins were detected after 24 h induction of their expression in recombinant bacteria, in terms of both immunospecificity and size (i.e., P450scc, 54 kDa (Fig. 3A); Adx, 12 kDa (Fig. 3B); and AdR, 51 kDa (Fig. 3C) corresponding to those of the adrenocortical P450scc system.

The content of cytochrome P450scc and its redox partners Adx, and AdR did not differ significantly between cells expressing P450scc system proteins and cells co-expressing these proteins and STARD1 or STARD3. Only a slight excess of Adx content is evident in the strain that does not synthesise transfer proteins (Fig. 3B, lane 2). However, the difference in the levels of P450scc redox partners present in significant amounts in cells is unlikely to affect the efficiency of P450scc function. As shown by densitometric analysis of immunospecific bands (*see Sect.* SDS-PAGE and Western immunoblotting) corresponding to the P450scc, main component of P450 system that catalyses the reactions of cholesterol transformation, the difference in its content in cells of the studied recombinant strains does not exceed 20% (data from 3 experiments). An example of calculating P450scc protein expression level is shown in Supplementary File. As stated above, each of the P450scc system proteins is formed by independent translation and it is likely that the presence of cDNAs encoding transfer proteins within the expression cassette does not significantly affect their expression levels.

### The effect of STARD1 and STARD3 on cholesterol hydroxylase/lyase activity of *E. coli* cells with the reconstructed P450scc system

To evaluate the impact of human START proteins on the cholesterol-transforming activity of *E. coli* cells incorporating the P450scc system, we compared the ability of cells synthesizing both P450 system proteins and a cholesterol transfer protein with that of cells synthesizing only P450 system proteins to carry out the biotransformation of cholesterol. In this study, non-transforming cells were used as the control strain.

**Fig. 4:**
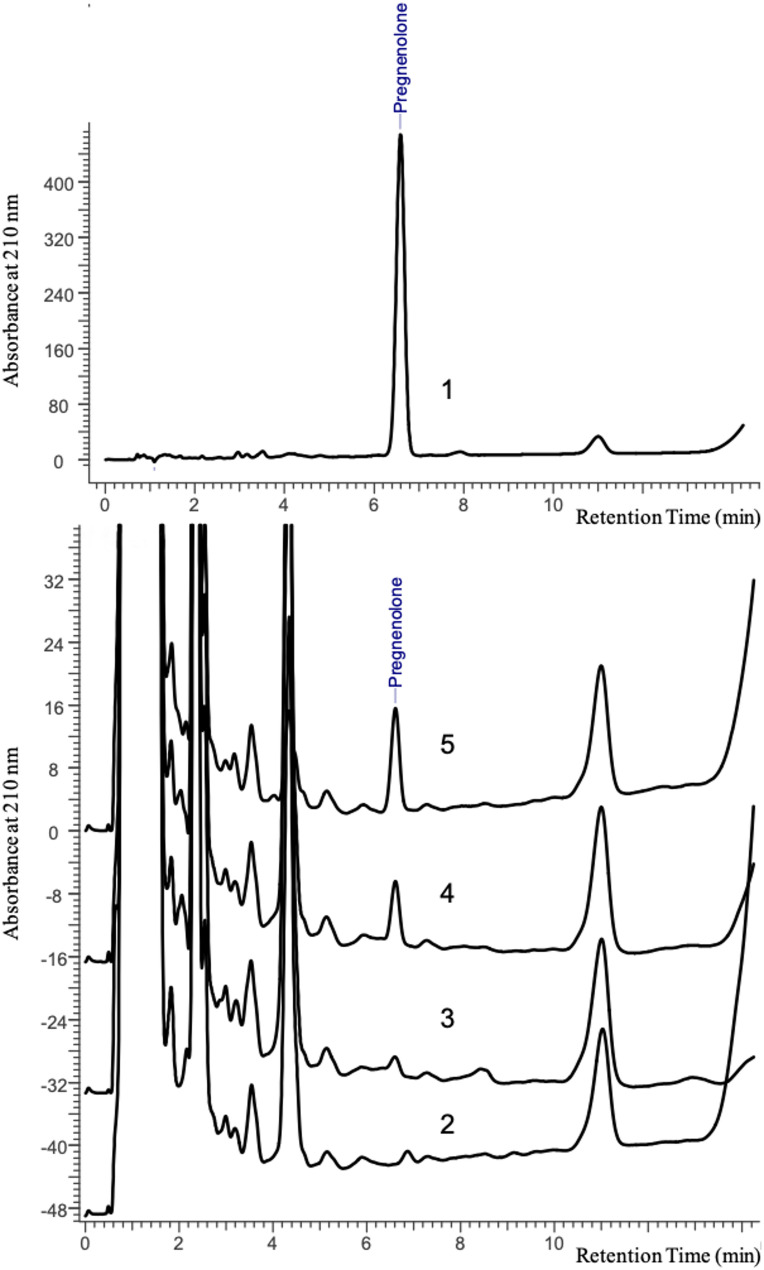
Reversed-phase HPLC analysis of organic extracts from the cultivation medium. 1, pregnenolone external standard injection (20 µl, 1 mg/ml). 2–5, chromatograms of the test samples (20 µl), obtained from cell cultures of control (2), recombinant *E. coli*/pET22b/CHL (3), *E. coli*/pET22b/STARD1‐CHL (4) and *E. coli*/pET22b/STARD3‐CHL (5) cells, respectively, incubated with cholesterol. Details of HPLC conditions are given in *Sect. *Measuring of cholesterol hydroxylase/lyase activity of *E. coli* cells in vivo

*E. coli* cells that had been transformed with plasmid pET22b/STARD1-CHL, pET22b/STARD3-CHL, or pET22b/CHL were cultivated as resting cells (concentrated cultures) in the presence of IPTG and the P450scc substrate cholesterol for 24 h. The formation of pregnenolone in recombinant cells was confirmed by HPLC analysis of samples obtained from cell cultures (*see Sect. *Measuring of cholesterol hydroxylase/lyase activity of *E. coli* cells in vivo for details of the experiments). Figure 4 shows fragments of HPLC chromatograms for a standard pregnenolone sample (Fig. 4, 1) as well as for samples under investigation (Fig. 4, 2–5), obtained in one of the experiments. A peak with a retention time of 6.6 min, corresponding to pregnenolone, was identified in organic extracts obtained from the culture medium of *E. coli*/pET22b/CHL, *E. coli*/pET22b/STARD1-CHL, and *E. coli*/pET22b/STARD3-CHL (Fig. 4, 3–5). However, this peak was not detected in extracts obtained from the culture medium of control *E. coli* cells cultured in the presence (Fig. 4, 2) or absence of cholesterol (control sample) (Fig. S4). As detected by HPLC, the pregnenolone content decreased in the samples in the following order: culture medium extract of *E. coli*/pET22b/STARD3-CHL (Fig. 4, 5), *E. coli*/pET22b/STARD1-CHL (Fig. 4), and *E. coli*/pET22/CHL (Fig. 4, 3).

In a subsequent series of experiments, an enzyme-linked immunosorbent assay (ELISA) was used to determine the amount of pregnenolone produced in recombinant cells (*see Sect. *Measuring of cholesterol hydroxylase/lyase activity of *E. coli* cells in vivo). The sterol content determined in cell culture preparations was then used to calculate the efficiency of cholesterol biotransformation by cells, which was expressed as mg of pregnenolone formed per liter of culture over 24 h (Tables S3, S4). In the presence of STARD1 and STARD3, the efficiency of cholesterol biotransformation by cells was increased at least 1.9-fold (95% CI [1.03–2.7]) and 3.2-fold (95% CI [1.4–4.9]), respectively, compared with that of *E. coli*/pET22b /CHL cells (the result of three experiments (three cultivations of these strains in parallel and at least five determinations of the content of the reaction product in the obtained samples)). Since the cells *E. coli*/pET22b/STARD1-CHL, *E. coli*/pET22b/STARD3-CHL, and *E. coli*/pET22b/CHL have similar levels of P450scc protein (*see Sect. *Co-expression of the STARD1 or STARD3 gene with genes of the P450scc system proteins), we can assume that the efficiency of pregnenolone synthesis correlates with STAR activity. This supports the hypothesis that cholesterol transporter proteins increase sterol accessibility for P450scc, thereby resulting in higher cholesterol conversion.

A comparison of two different members of the START lipid transfer protein family, STARD1 and STARD3, synthesised in *E. coli* cells, in terms of their level of synthesis, stability and effect on the activity of the P450scc system, showed that these proteins exhibit different characteristics, and that STARD3 being the more promising sterol carrier into bacterial cells (Table S4). The maximum efficiency of cholesterol conversion in *E. coli*/pET22b/STARD3-CHL cells, as determined by ELISA, was 5.96 mg of pregnenolone per 24 h per liter of culture (Table S3). It the course of the work, we found also that pregnenolone produced in the cells is secreted into the culture medium (data not shown). In the case of using bacteria to produce of steroid compounds, this greatly simplifies the process of isolating the desired steroid.

Pregnenolone and the steroid hormones derived from it have a wide range of applications in various fields of medicine. Consequently, there is a great deal of interest in research aimed at engineering strains of various microorganisms with enhanced P450scc catalytic activity for use in pregnenolone biosynthesis. Such works have been carried out in a number of laboratories (e.g. see Szczebara et al. 2003; Mauersberger et al. 2013; Gerber et al. 2015; Efimova et al. 2019b; Zhang et al. 2019; Karpov et al. 2024). However, new recombinant strains and approaches are still needed to produce this widely demanded steroid in high yield.

In this study, we used the bacterium *E. coli*, a well-studied and “simpler” organism for use in biotechnological processes (Hanlon et al. 2007). When using *E. coli* cells, there is a wide choice of strains, plasmid vectors, and protocols developed for the expression of heterologous genes. The absence of its own cytochrome P450 enzymes makes *E. coli* a convenient host for the reconstruction of recombinant P450 systems (Nelson et al. 1993). Moreover, they can synthesize mature forms of mammalian mitochondrial and microsomal P450s in a functionally active state, reducing the number of steps required for topogenesis to form catalytically active proteins (Novikova et al. 2009). We have obtained a *E. coli*/pET22b/STARD3-CHL bacterial strain co-expressing STARD3 and P450scc system proteins. This strain exhibits productivity (up to ~ 6 mg/L), which is not yet sufficient to utilize the bacteria for biotechnological purposes. However, microorganisms capable of performing processes occuring in the human body, even those that do not exhibit high activity, are interesting for conducting research as alternative model systems for animal experiments and as simple test systems for screening drug compounds for their activity and toxicity.

In general, in the paper we have demonstrated, for the first time, that the synthesis of a specific cholesterol carrier in *E. coli* can enhance sterol absorption by cells, and that this property can be exploited to optimize the functioning of the P450 cholesterol-hydroxylating system in cells. Moreover, the findings of this study indicate the possibility of investigating alternative proteins to enhance the efficiency of steroid transport into *E. coli* cells. For example, in addition to STARD1 and STARD3, other members of the STARD family of lipid transfer proteins belonging to the STARD4 subfamily (forms 4–6) (Larsen et al. 2020), as well as yeast Lam/Ltc proteins and human GRAMDs (Aster-A, -B and -C) containing START-like domains (Elustondo et al. 2017), are capable of transporting cholesterol across cell membranes. In addition, ABC family proteins have the ability to transport cholesterol and other steroid compounds across membranes. They are found both in mammals (e.g. the transporters ABCA1 and ABCG1 (Yañez and Leiva 2022) and in certain microorganisms, such as mycobacteria (Mohn et al. 2008) and the yeast *Y. lipolytica* (Thevenieau et al. 2007). In one study, the expression of ABC1 and ABC2 *Y. lipolytica* transporters in *S. cerevisiae* cells was successfully used to rapidly remove toxic organic compounds from the cells during biofuel production, resulting in increased cell tolerance, longevity, and product yield (Chen et al. 2013). However, to our knowledge, there are currently no studies in the literature that utilize STAR proteins or ABC transporters to enhance the uptake of hydrophobic compounds by cells.

## Conclusions

The present study demonstrates the biological activity of recombinant STARD3 in *E. coli*. We show that human STARD3, similar to human STARD1 (Zamalutdinova et al. 2022), acts as a lipid transporter protein and promotes the transfer of sterols into intact *E. coli* cells. Our work is the first to explore this aspect of the behavior of cholesterol carrier proteins in a bacterial host. Using an in vivo combination of a cholesterol transporter protein, either STARD3 or STARD1, with a reconstituted P450scc system in *E. coli* cells, we found that, in the presence of a cholesterol transporter protein, pregnenolone synthesis in the target strains increased by at least 3.2-fold and 1.9-fold, respectively. Overall, our research represents a novel approach to optimizing the function of the steroidogenic system in the microorganisms by using of a specific carrier protein in order to overcome the significant challenge of low microbial sterol conversion - low penetration efficiency of poorly soluble steroid compounds into the cells. The results of this study can be used as a starting point for future investigation focused at understanding the mechanisms of cholesterol transfer proteins functioning in *E. coli* cells and further development of this approach, which could be applied in the preparation of microbial biocatalysts.

## Electronic supplementary material

Below is the link to the electronic supplementary material.

Supplementary Material 1

Supplementary Material 2

## Data Availability

All data generated during this study are included in this published article.

## Abbreviations

P450scc: cytochrome P450 cholesterol hydroxylase/20,22-lyase (CYP11A1)
Adx: Adrenodoxin
AdR: NADPH-adrenodoxin reductase
NBD: Nitrobenzoxadiazole
20NP: 20-(NBD)amino-pregn-5-en-3β-ol
GFP: Green fluorescent protein
MCD: Methyl-β-cyclodextrin
STARD1: Steroidogenic acute regulatory protein
STARD3: MLN64,StAR-related lipid transfer protein-3
