# Supplementary material for: Using of transporter proteins to improve the uptake efficiency of hydrophobic compounds by Escherichia coli: a coordinated synthesis of START protein and P450scc system proteins to enhance cholesterol biotransformation
Source: Bioresour Bioprocess. 2025 Jul 21;12(1):77. doi: 10.1186/s40643-025-00909-1 (PMC12279672; doi:10.1186/s40643-025-00909-1)
Supplement: Supplementary file 1 — Supplementary Material 1 [file 40643_2025_909_MOESM1_ESM.docx]

**Supplementary Information**

**
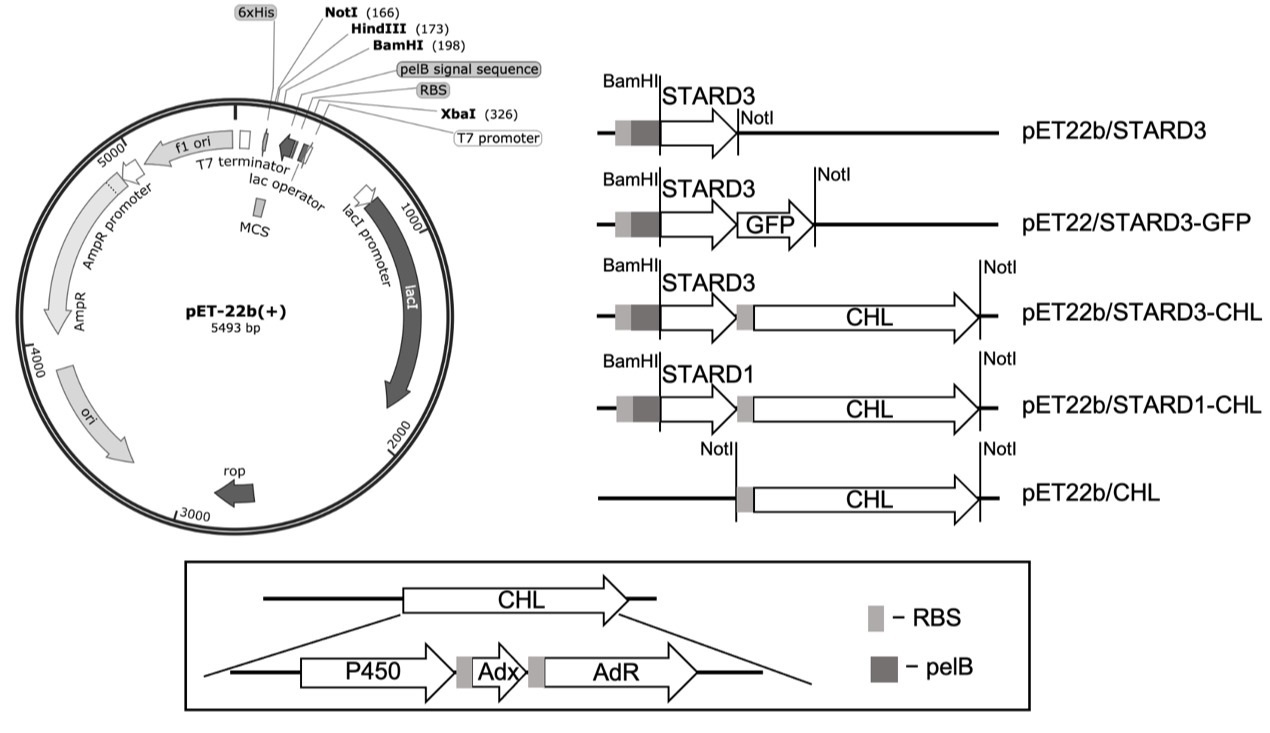
**

**Supplementary Figure S1.** Diagram of the base plasmid pET-22b(+) (left) and schematic representation of the arrangement of cDNAs for heterologous proteins in the expression cassettes of the constructed polycistronic plasmids (right). The cDNA encoding P450scc-RBS-Adx-RBS-AdR is designated CHL.

**Supplementary Table S1.** Oligonucleotides used in work.

| Oligonucleotide  number | Oligonucleotide sequence 5′-3′  (added restriction enzyme sites are marked with italic bold) | Restriction sites |
| --- | --- | --- |
| 1 | GTTTCA***GGATCC***GGGCTCTGATAACG | *BamH*I |
| 2 | GCGGCCGC***TCTAGA***TGCACCC | *Xba*I |
| 3 | GGGCCT***TCTAGA***ATGG | *Xba*I |
| 4 | CGAGGCTGA***GCGGCCGC***TTTAATTC | *Not*I |
| 5 | TGTGA***GCGGCCGC***CAATTTCACACAGGAAAC | *Not*I |
| 6 | CTAGAGGATCCC***GCGGCCGC***TCAGTGCCCCAGCAGC | *Not*I |
| 7 | TGCTGAGCAGCATTGGGTAT | - |
| 8 | CAATGATACCGCGAGACCCA | - |

**Supplementary Table S2.** The conditions of the experiments carried out in this study.

| **Investigated parameter (Experiment)** | **Temperature (°C)** | **Shaking (rpm)** | **Induction medium** | **Transcription inducer** | **Time of induction (h)** |
| --- | --- | --- | --- | --- | --- |
| GFP-STAR fluorescence | 25 | 180 | TB | IPTG | 17 |
| Content of proteins (Western blotting, anti-GFP) | 25 | 180 | TB | IPTG | 17 |
| STAR functional activity (substrate-NBD) | 25 | 180 | Autoinduction medium | lactose | 24-48 |
| STAR functional activity (substrate-NP) | 25 | 180 | Autoinduction medium | lactose | 24-48 |
| STAR functional activity (substrate-cholesterol) | 25 | 180 | TB | IPTG | 24 |
| Content of proteins (Western blotting, anti-P450scc/Adx/AdR) | 25 | 180 | TB | IPTG | 24 |
| CHL activity (substrate-cholesterol) | 25 | 180 | TB; NaP buffer (for resting cells) | IPTG | 24; 24 (resting cells) |
|  | 37 | 200 | Autoinduction medium | lactose | 24 |

See the “Materials and methods” for details.


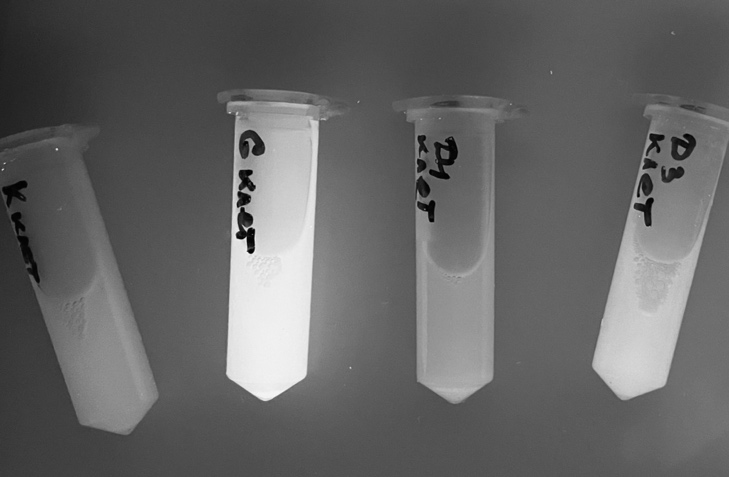


1 2 3 4

**Supplementary Figure S2**. Photograph under UV illumination of cells taken after cultivation under induction conditions. Fluorescence of suspensions of: non-transformed *E. coli* cell (1), *E. coli* cells, transformed with pET22b/GFP (2), pET22b/STARD1-GFP (3), or pET22b/STARD3-GFP (4). The suspensions were in 50 mM Tris-HCl and had an А_600_ = l5. Illumination with UV light of 366 nm.

**A**

| **Sample Name** | 7x100 **Control** | **Sample Type** | Unknown |
| --- | --- | --- | --- |
| **Acquisition Method** | Cholesterol_Isocratic.dam | **Algorithm Used** | MQ4 |
| **Project** | Cholesterol | **Instrument Name** | QTRAP 4500 |
| **Data File** | Cholesterol_S2.wiff | **Result Table** | CholesterolS2 |

| \| Cholesterol 2 (369.1 / 135.0) \| \| \| \| --- \| --- \| --- \| \|  \| \| \| \| RT (Exp. RT): \| 1.58 (1.57) min \| \| \| Calculated conc: \| Degenerateng/ml \| \| \| Analyte Area: \| 442449 \|  \| \| Sample Type: \| (Unknown) \| \| | 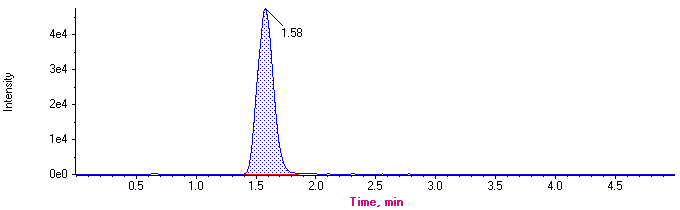 |
| --- | --- | --- | --- | --- | --- | --- | --- | --- | --- | --- | --- | --- | --- | --- | --- | --- | --- | --- | --- |

| \| Cholesterol 4 (369.1 / 161.2) \| \| \| \| --- \| --- \| --- \| \|  \| \| \| \| RT (Exp. RT): \| 1.58 (1.57) min \| \| \| Calculated conc: \| **518043.45 ng/ml** \| \| \| Analyte Area: \| 781388 \|  \| \| Sample Type: \| (Unknown) \| \| | 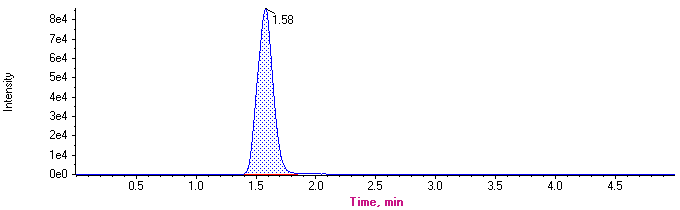 |
| --- | --- | --- | --- | --- | --- | --- | --- | --- | --- | --- | --- | --- | --- | --- | --- | --- | --- | --- | --- |

**B**

| **Sample Name** | 9x100 **STARD1** | **Sample Type** | Unknown |
| --- | --- | --- | --- |
| **Acquisition Method** | Cholesterol_Isocratic.dam | **Algorithm Used** | MQ4 |
| **Project** | Cholesterol | **Instrument Name** | QTRAP 4500 |
| **Data File** | Cholesterol_S2.wiff | **Result Table** | CholesterolS2 |

| \| Cholesterol 2 (369.1 / 135.0) \| \| \| \| --- \| --- \| --- \| \|  \| \| \| \| RT (Exp. RT): \| 1.58 (1.57) min \| \| \| Calculated conc: \| degenerateng/ml \| \| \| Analyte Area: \| 711145 \|  \| \| Sample Type: \| (Unknown) \| \| | 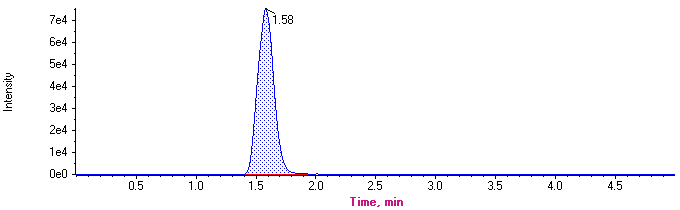 |
| --- | --- | --- | --- | --- | --- | --- | --- | --- | --- | --- | --- | --- | --- | --- | --- | --- | --- | --- | --- |

| \| Cholesterol 4 (369.1 / 161.2) \| \| \| \| --- \| --- \| --- \| \|  \| \| \| \| RT (Exp. RT): \| 1.58 (1.57) min \| \| \| Calculated conc: \| **837382.83 ng/ml** \| \| \| Analyte Area: \| 1261904 \|  \| \| Sample Type: \| (Unknown) \| \| | 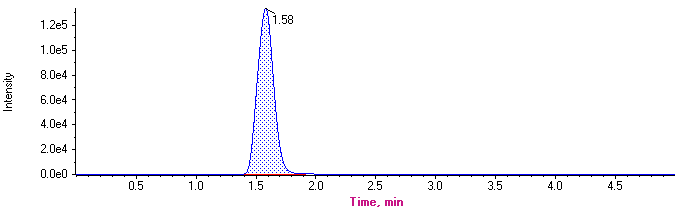 |
| --- | --- | --- | --- | --- | --- | --- | --- | --- | --- | --- | --- | --- | --- | --- | --- | --- | --- | --- | --- |

**C**

| **Sample Name** | 10x100 **STARD3** | **Sample Type** | Unknown |
| --- | --- | --- | --- |
| **Acquisition Method** | Cholesterol_Isocratic.dam | **Algorithm Used** | MQ4 |
| **Project** | Cholesterol | **Instrument Name** | QTRAP 4500 |
| **Data File** | Cholesterol_S2.wiff | **Result Table** | CholesterolS2 |

| \| Cholesterol 2 (369.1 / 135.0) \| \| \| \| --- \| --- \| --- \| \|  \| \| \| \| RT (Exp. RT): \| 1.58 (1.57) min \| \| \| Calculated conc: \| Degenerateng/ml \| \| \| Analyte Area: \| 869658 \|  \| \| Sample Type: \| (Unknown) \| \| | 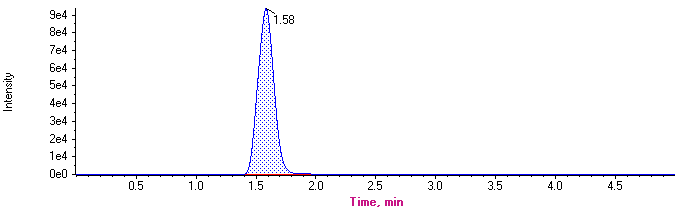 |
| --- | --- | --- | --- | --- | --- | --- | --- | --- | --- | --- | --- | --- | --- | --- | --- | --- | --- | --- | --- |

| \| Cholesterol 4 (369.1 / 161.2) \| \| \| \| --- \| --- \| --- \| \|  \| \| \| \| RT (Exp. RT): \| 1.58 (1.57) min \| \| \| Calculated conc: \| **1016035.88 ng/ml** \| \| \| Analyte Area: \| 1530726 \|  \| \| Sample Type: \| (Unknown) \| \| | 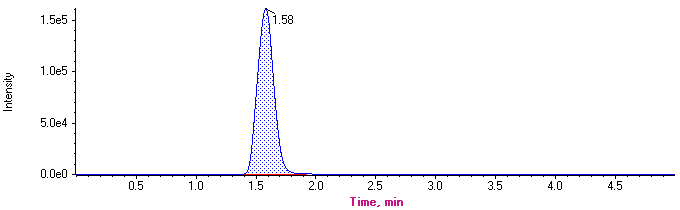 |
| --- | --- | --- | --- | --- | --- | --- | --- | --- | --- | --- | --- | --- | --- | --- | --- | --- | --- | --- | --- |

**Supplementary Figure S3**. The results of an experiment to determine the cholesterol content in untransformed (control) *E. coli* BL21(DE3) cells (**A**) and in recombinant *E.coli*/pET22b/STARD1-CHL (**B**) and *E.coli*/pET22b/STARD3-CHL (**C**) cells, cultured in the presence of cholesterol for 23 hours, using HPLC-MS. For details on the experiment, see the "Materials and Methods" (*section 2.7*).

**
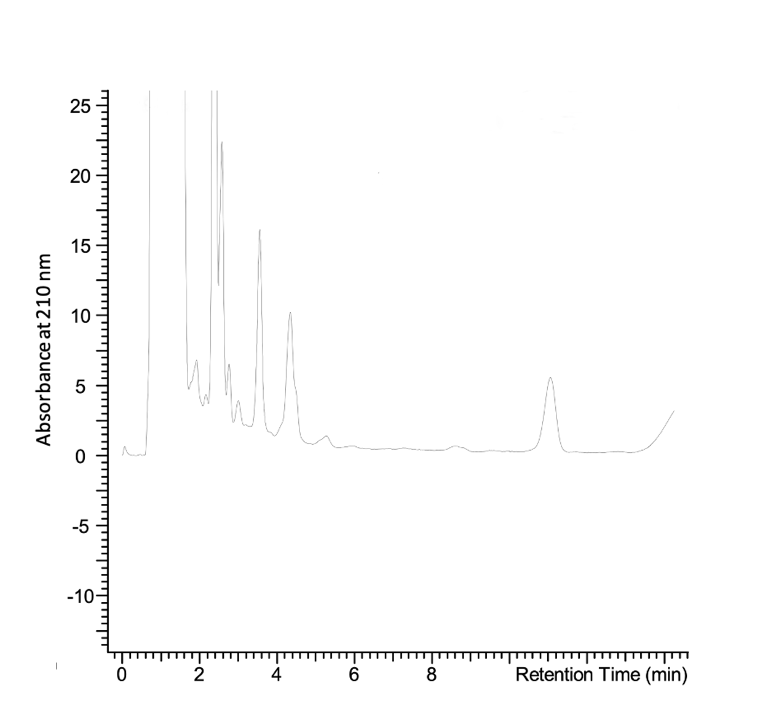
**

**Supplementary Figure S4**. Reversed‐phase HPLC analysis of organic extract (20 μl) from the cultivation medium obtained from cell culture of control *E.coli* cells incubated without cholesterol. Details of HPLC conditions are given in *section “Measuring of cholesterol hydroxylase/lyase activity of E. coli cells in vivo”.*

**Supplementary Table S3.** The influence of human cholesterol transfer proteins, STARD1 and STARD3, on the cholesterol-transforming activity of *E. coli* cells incorporating the P450scc system. Activity measured in preparations obtained from cultures of *E.coli* cells using ELISA. Details of the determination of activity are given in *section “Measuring of cholesterol hydroxylase/lyase activity of E. coli cells in vivo”*.

| **Plasmid used for cell transformation** | **Activity**  **(mg of pregnenolon/L of culture x 24 hours)** |
| --- | --- |
| pET22b/CHL | 1.86 |
| pET22b/STARD1-CHL | 3.5 |
| pET22b/STARD3-CHL | 5.96 |

The table shows the mean values of three experiments (three cultivations of the indicated strains in parallel, and at least five determinations of the content of the reaction product in the obtained samples).

**Supplementary Table S4.** Comparison of characteristics of STARD1 and STARD3 in *E.coli* cells.

| **Investigated parameter** | **Non-transformed (control) *E. coli* cells** | **Ratio of content or effect** | |
| --- | --- | --- | --- |
|  |  | **STARD1** | **STARD3** |
| GFP-STAR fluorescence | - | 1 | 3±0.6* |
| Content of protein (Western blotting, anti-GFP) | - | 1 | 8±3* |
| STAR functional activity (substrate - NBD) | 1 | 1.3(95%C.I. [1.1-1.5]) | 1.5 (95%C.I. [1.2-1.8]) |
| STAR functional activity (substrate - NP) | 1 | 1.5 (95%C.I. [0.9-2.1]) | 3 (95%C.I. [2.1-4.0]) |
| STAR functional activity (substrate - cholesterol) | 1** | 1.34±0.7* | 1.62±0.28* |
|  | ***E. coli* cells transformed with pET22b/CHL** |  |  |
| CHL activity (substrate - cholesterol) | 1*** | 1,9 (95% C.I. [1.03-2.7]) | 3.2 (95% C.I. [1.4-4.8]) |

* - Mean ± SD; p < 0.05.

** - The average cholesterol content measured in untransformed *E.coli* cells after 24 hours of growth in the presence of cholesterol (46 micrograms of cholesterol per number of cells in the 1 ml of culture at an optical density of 1.0), was taken as 1.

*** - The average CHL activity measured in *E. coli*/pET22b/CHL cells after 24 hours of growth in the presence of cholesterol (1.86 mg of pregnenolone per litre of culture), was taken as 1.
