# Supplementary material for: Using of transporter proteins to improve the uptake efficiency of hydrophobic compounds by Escherichia coli: a coordinated synthesis of START protein and P450scc system proteins to enhance cholesterol biotransformation
Source: Bioresour Bioprocess. 2025 Jul 21;12(1):77. doi: 10.1186/s40643-025-00909-1 (PMC12279672; doi:10.1186/s40643-025-00909-1)
Supplement: Supplementary file 2 — Supplementary Material 2 [file 40643_2025_909_MOESM2_ESM.docx]

**Supplementary file**

**Full uncropped blot images for Fig. 1B and Fig.3.**


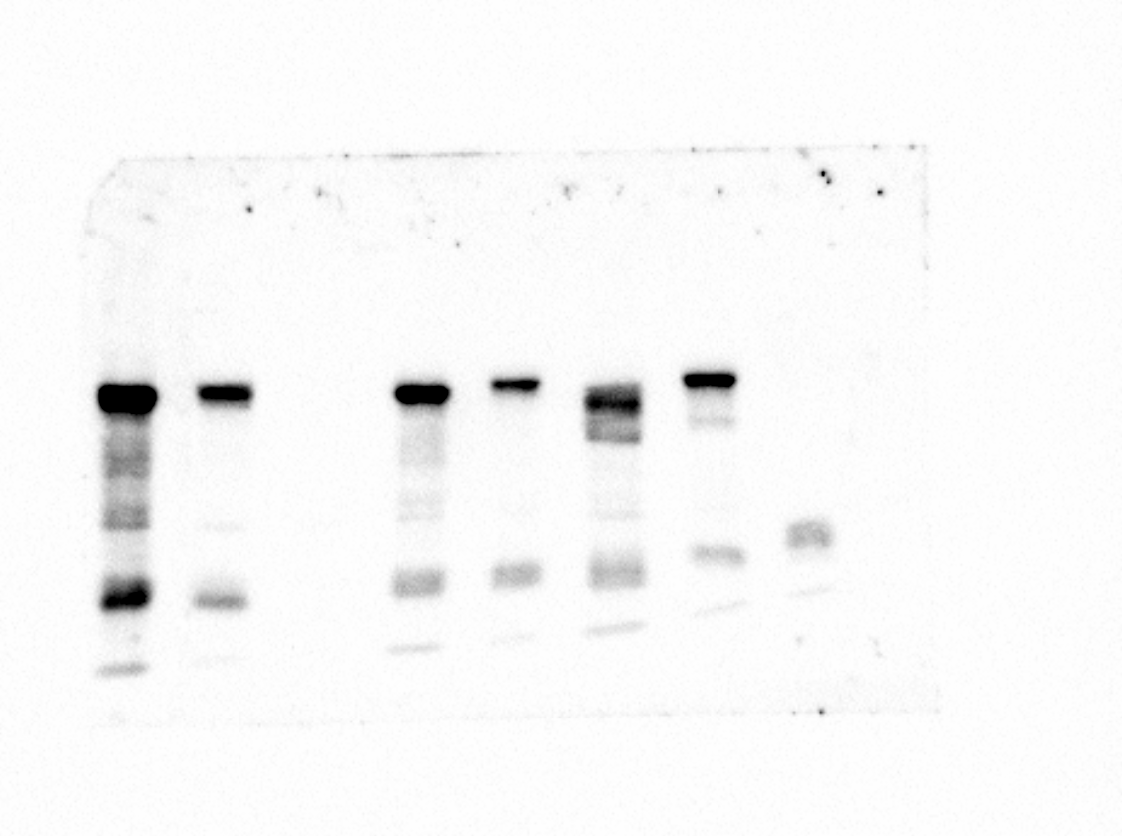


STARD3-GFP

STARD1-GFP

4 3 2 1

kDa

26

34

43

55

1 2 3 4 5 6 7 8 9

**The numbers located at the top correspond to the lane numbers in Fig. 1B.**

**Figure 1B.** SDS-PAGE (10% gel) and Western blotting analysis using anti-GFP antibodies; lanes: 1, control cell lysate (10 μg); 2, GFP standard (0.25 μg); 3, *E. coli*/pET22b/STARD1-GFP cell lysate (10 μg); 4, *E. coli*/pET22b/STARD3-GFP cell lysate. (1.5 μg). The molecular weights of standard protein markers (kDa) are shown on the left.

**The numbers located at the bottom correspond to the lane numbers of the full uncropped blot.** For information according lines 3, 4, 6, 8 see legend of Fig. 1B.

1 – *E. coli*/pET22b/STARD3-GFP cell lysate (10 μg);

**2** – *E. coli*/pET22b/T-D3-GFP cell lysate (10 μg);

**5** – *E. coli*/pET22b/T-D3-GFP cell lysate (5 μg);

**7** – *E. coli*/pET22b/T-D1-GFP cell lysate (2,5 μg);

9 – control cell lysate (10 μg)

*E. coli* strains *E. coli*/pET22b/T-D1-GFP and *E. coli*/pET22b/T-D3-GFP don’t concern to present study.

**B**

**C**

**A**

kDa


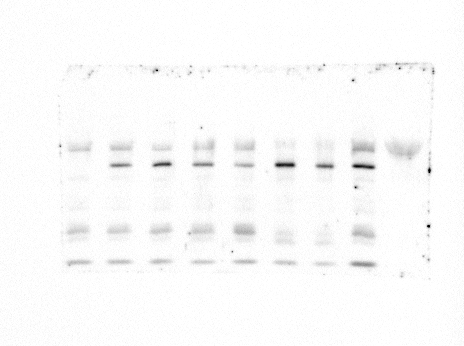

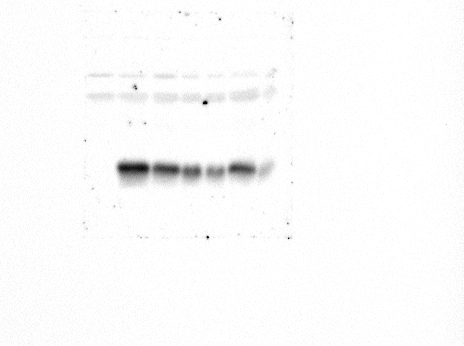

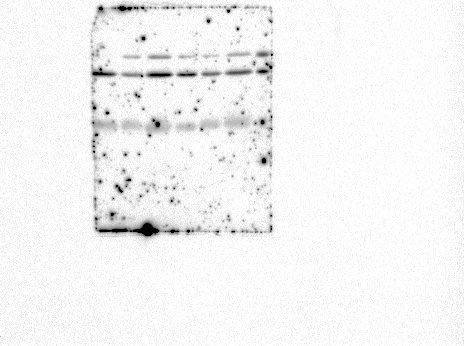


26

**D**

P450scc

AdR

Adx

kDa

kDa

10

55

55

1 2 3 4 5

1 2 3 4 5 6 7

1 2 3 4 5

1 2 3 4 5 6 7 8 9

1 2 3 4 5 6 7

1 2 3 4 5

| Lane | Intracellular protein with a molecular weight of 26 kDa (c. u.) | Times (coefficient) | P450scc (c. u.) | P450scc (c. u.) x coefficient | Content of P450scc (%) |
| --- | --- | --- | --- | --- | --- |
| 1 | 102 250 891 062 |  | 13 356 909 557 |  |  |
| 2 | 94 425 615 674 | 1 | 141 764 767 443 | 141 764 767 443 | 100 |
| 3 | 102 149 614 704 | 1.08 | 178 223 203 621 | 165 021 484 834 | 116.41 |
| 4 | 111 891 260 111 | 1.18 | 156 608 594 297 | 132 719 147 709 | 93.62 |

**The numbers located at the top correspond to the lane numbers in Fig. 3.**

**Figure 3.** Co-expression of bovine P450scc, Adx, and AdR in IPTG-induced *E. coli* cells, transformed with pET22b/CHL (2), pET22b/STARD1-CHL (3), or pET22b/STARD3-CHL (4). Western blotting of cell lysates after SDS-PAGE in 10% (A) or 15% (B, C) gel followed by immunostaining with antibodies against P450scc (*A*), Adx (*B*), or AdR (*C*). Non-transformed *E. coli* cell lysate (1) was used as a negative control. Lysate of *E. coli*/pBar_Triple cells (Efimova et al.,2019a) (5) synthesizing three separate mature proteins of the P450scc system was used as positive control. Total protein loading/lane: *A*, lines 1-5: 3 μg, respectively; *B*, lines 1-4, 5: 60 μg and 20 μg, respectively; *C*, lines 1-4, 5: 30 μg and 20 μg, respectively. The closest molecular weight of standard protein markers (kDa) is shown on the left.

(D) – an example of calculating P450scc protein expression level using densitometry data and loading control data (Intracellular protein with a molecular weight of 26 kDa) to normalize the amount of cellular protein plated on a gel lines.

**The numbers located at the bottom correspond to the lane numbers of the full uncropped blot.** For information according lines 1-4, 6 see legend of Fig. 3.

A: 5 – *E. coli*/pTrc_D3_CHL (3 μg); 7 – *E. coli*/pBar_Triple (1 μg); 8 – *E. coli*/pET_CHL (6 μg), 9 – a sample from another study

B: 4 – *E. coli*/pET_D1_CHL (30 μg), 5 – *E. coli*/pET_D3_CHL (30 μg)

C: 4 – *E. coli*/pET_D1_CHL (60 μg), 5 – *E. coli*/pET_D3_CHL (60 μg)


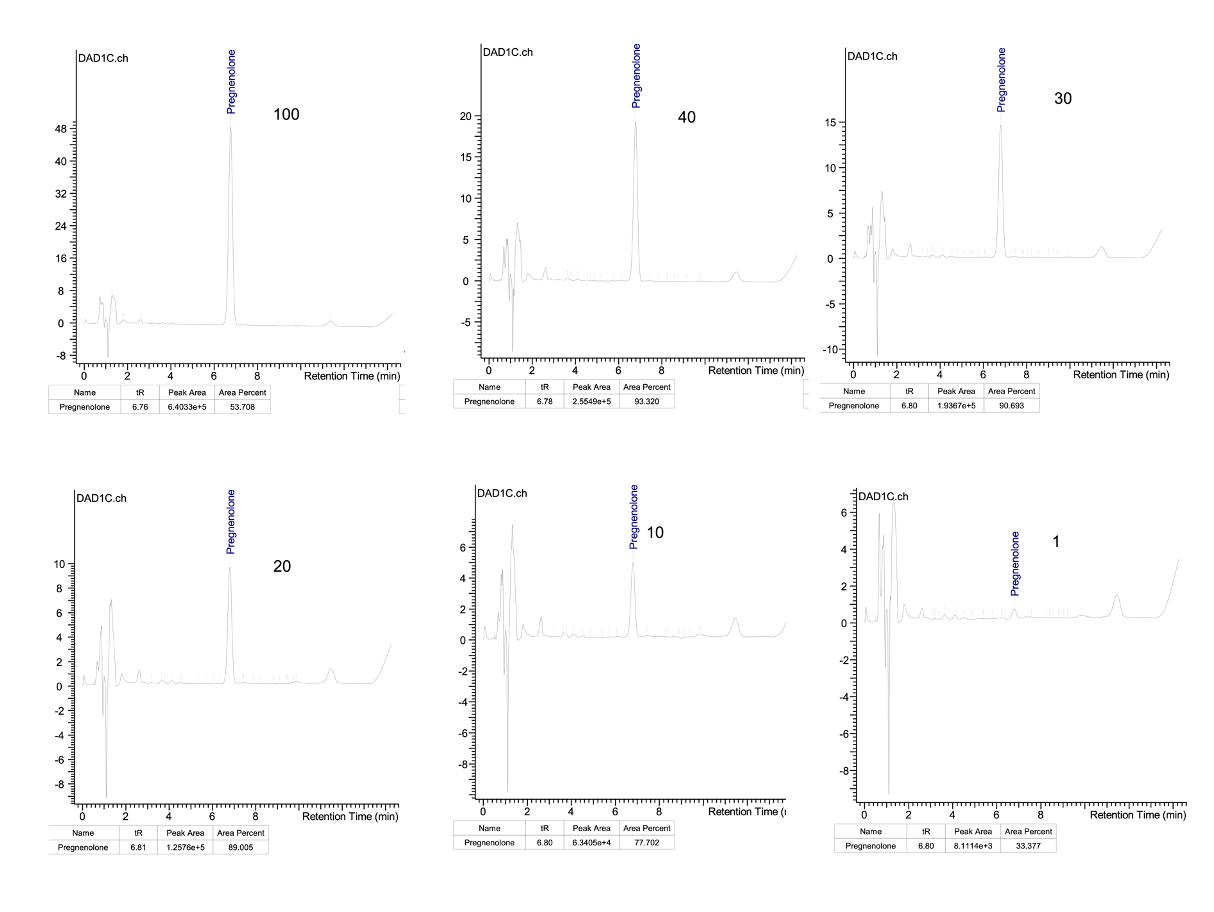


Reversed‐phase HPLC analysis of standard pregnenolone solutions in 96% EtOH. 1-6, pregnenolone external standard injection (20 µl, 100, 40, 30, 20, 10, 1 μg /ml), used to obtain a calibration curve during HPLC analysis of the pregnenolone content in experimental samples.
